# Oligodendrocyte Development in the Absence of Their Target Axons *In Vivo*

**DOI:** 10.1371/journal.pone.0164432

**Published:** 2016-10-07

**Authors:** Rafael Almeida, David Lyons

**Affiliations:** 1 Centre for Neuroregeneration, University of Edinburgh, Edinburgh, United Kingdom; 2 MS Society Centre for Translational Research, University of Edinburgh, Edinburgh, United Kingdom; 3 Euan MacDonald Centre for Motor Neurone Disease Research, University of Edinburgh, Edinburgh, United Kingdom; Massachusetts General Hospital/Harvard Medical School, UNITED STATES

## Abstract

Oligodendrocytes form myelin around axons of the central nervous system, enabling saltatory conduction. Recent work has established that axons can regulate certain aspects of oligodendrocyte development and myelination, yet remarkably oligodendrocytes in culture retain the ability to differentiate in the absence of axons and elaborate myelin sheaths around synthetic axon-like substrates. It remains unclear the extent to which the life-course of oligodendrocytes requires the presence of, or signals derived from axons in vivo. In particular, it is unclear whether the specific axons fated for myelination regulate the oligodendrocyte population in a living organism, and if so, which precise steps of oligodendrocyte-cell lineage progression are regulated by target axons. Here, we use live-imaging of zebrafish larvae carrying transgenic reporters that label oligodendrocyte-lineage cells to investigate which aspects of oligodendrocyte development, from specification to differentiation, are affected when we manipulate the target axonal environment. To drastically reduce the number of axons targeted for myelination, we use a previously identified kinesin-binding protein (*kbp*) mutant, in which the first myelinated axons in the spinal cord, reticulospinal axons, do not fully grow in length, creating a region in the posterior spinal cord where most initial targets for myelination are absent. We find that a 73% reduction of reticulospinal axon surface in the posterior spinal cord of *kbp* mutants results in a 27% reduction in the number of oligodendrocytes. By time-lapse analysis of transgenic OPC reporters, we find that the reduction in oligodendrocyte number is explained by a reduction in OPC proliferation and survival. Interestingly, OPC specification and migration are unaltered in the near absence of normal axonal targets. Finally, we find that timely differentiation of OPCs into oligodendrocytes does not depend at all on the presence of target axons. Together, our data illustrate the power of zebrafish for studying the entire life-course of the oligodendrocyte lineage in vivo in an altered axonal environment.

## Introduction

Oligodendrocytes form the myelin sheaths of the central nervous system (CNS) which insulate axons and enable fast propagation of action potentials [1]. Myelinating oligodendrocytes also provide axons with metabolic support [2, 3]. Many CNS axons become myelinated over time [1, 4], and myelin sheaths seem exquisitely adjusted to each axon in length [4] and thickness [5], potentially to optimize conduction velocity for the particular circuit in which they are integrated [6–9]. These observations suggest that axons ought to play a key role in regulating oligodendrocyte development and myelination. In fact, seminal studies in the optic nerve indicated that retinal ganglion cell axons regulate oligodendrocyte precursor cell (OPC) proliferation and oligodendrocyte survival [10–15], and recently, optogenetic stimulation of cortical neurons was shown to increase OPC proliferation and myelination [16, 17]. Increasing neuronal activity in the zebrafish spinal cord leads to an increase in oligodendrocyte number and stimulates oligodendrocytes to make more sheaths [18], while inhibiting synaptic vesicle release had the opposite effect [18, 19]. Indeed, the presence of supernumerary large-diameter axons fated for myelination in the zebrafish CNS is enough to induce oligodendrocytes to elaborate more myelin sheaths [20]. Thus, axons can regulate specific aspects of oligodendrocyte development and myelination in vivo. Remarkably, however, oligodendrocytes can also fully differentiate and form myelin in the virtual absence of axons in vitro [21–24]. Over thirty years ago, it was found that OPCs dissociated from brain [25] or optic nerve [26] retained the ability to differentiate on schedule regardless of the age at dissociation, or presence of neurons or axons [21]. Additional studies further illustrated neuron-independent developmental programmes, whereby the daughter cells of individual OPCs, cultured in separate microwells, divide the same number of times and differentiate simultaneously [27]. As long as platelet-derived growth factor and thyroid hormone are present in the culture medium, dissociated OPCs are able to proliferate and, similar to OPCs in vivo, progressively mature in morphology and antigenic phenotype [28, 29]. They exit the cell cycle in a timely manner and start differentiating: they extend many ramified processes [30, 31]; start expressing myelin structural proteins and glycolipids [21, 32], and even form flat so-called “myelin sheets” on the petri dish surface [33–35]. Remarkably, recent studies have shown that oligodendrocytes can even elaborate myelin sheaths around inert plastic fibers of axon-like dimensions or formaldehyde-fixed axons [23, 24, 36–38], bringing into question the necessity of axons for CNS myelination per se.

Therefore, our understanding of the extent to which the distinct stages of oligodendrocyte development in vivo are regulated by axons is not clear, because it has not been possible to follow their development in its entirety over time, within one system, while simultaneously manipulating the axonal environment in the CNS. In particular, how would oligodendrocyte development be affected if the specific axons that are targeted for myelination in the CNS were absent in an otherwise normal axonal environment?

Here we use zebrafish to follow oligodendrocyte development from specification to differentiation and myelination in vivo. To disrupt axons that are fated for myelination, we focus on reticulospinal axons in the spinal cord. Reticulospinal neurons reside in the midbrain and hindbrain and project large-diameter axons along the entire length of the ventral spinal cord, where they are the first to be myelinated [20]. Given their stereotyped position and large calibre, reticulospinal axons are readily identifiable. To perturb their development, we use a previously identified *kinesin-binding protein* (*kbp*) mutant, in which the outgrowth of reticulospinal axons is specifically disrupted due to neuron-autonomous microtubule disorganization [39]. In the posterior (caudal) end of the spinal cord of *kbp*^st23^ mutants, reticulospinal axons are essentially absent, allowing us to study oligodendrocyte development and myelination in the near absence of their normal “correct” axonal targets. We find that early OPC specification and migration do not require the presence of correct axonal targets *in vivo*. We further show that when their correct targets are greatly reduced, the low basal level of OPC proliferation is further reduced, and oligodendrocyte survival is clearly impaired, in agreement with multiple roles for axons in regulating oligodendrocyte-lineage cell development. Importantly, in the anterior (rostral) spinal cord, where the reduction in reticulospinal axons is less drastic, oligodendrocyte-lineage cells develop normally, indicating that this effect is not due to the global disruption to *kbp*. Finally, we find that in the absence of correct target axons, oligodendrocytes retain the capacity to differentiate on time, highlighting that only some aspects of oligodendrocyte-lineage cell development are regulated by their target axons.

## Materials and Methods

### Fish husbandry and lines used

All animals used in this study were maintained under standard conditions [40, 41] in the Queen’s Medical Research Institute zebrafish facility. All animal studies were carried out with approval from the UK Home Office and according to its regulations, under project licenses 60/8436 and 70/8436. Adult animals were kept in a 14 hours light and 10 hours dark cycle. Embryos were kept at 28.5°C in 10mM HEPES-buffered E3 Embryo medium or conditioned aquarium water with methylene blue. Embryos were staged according to Kimmel et al (1995) [42]. The following zebrafish mutant or transgenic lines were used: *kbp*^st23^ [39]; Tg(olig2:EGFP) [43]; Tg(sox10:mRFP) [44]; Tg(mbp:EGFP) [20]; Tg(sox10:KalTA4) [45] and Tg(UAS:kaede) [46]. All transgenic lines were crossed into the *kbp*^st23^ mutant line. Throughout the text including figures, ‘Tg’ denotes a stable, germline inserted transgenic line.

### *kbp*^st23^ genotyping

Animals were genotyped for *kbp*^st23^ according to [39]: briefly, genomic DNA was extracted by standard protocols [47], from which a 250bp long PCR product was amplified using primers 5′-AAAACGACCAACTGTGCCTA-3′ and 5′-ACAGTCAAACACCAGATCGAAAGTCA-3′, and digested with restriction enzyme MaeIII (Roche) or NmuCI (Thermo Scientific). The WT PCR product is cleaved into 228bp and 22bp fragments; the mutant PCR product is not cleaved. In some experiments, *kbp*^st23^ homozygous mutants were identified by direct visualization of an incompletely grown posterior lateral line and ventral reticulospinal tract in the spinal cord in Tg(sox10:mRFP) larvae or 3A10 immunostained larvae. Unless otherwise stated, mutants were compared to wildtype-siblings (“WT”).

### Acridine Orange treatment

Dechorionated larvae were incubated for 30 minutes in the dark in 10mM HEPES-buffered E3 Embryo medium containing 2pg/ml acridine orange (Sigma), followed by two 5-minute washes in embryo medium, and immediately live-imaged after.

### Antibody labeling

Whole-mount antibody labelling was carried out using standard protocols [47]. Briefly, zebrafish were terminally anaesthetised in tricaine (3-amino benzoic acid ethyl ester, Sigma) and incubated immediately in primary fixative (2% paraformaldehyde/ 1% trichloroacetic acid, or 4% paraformaldehyde). Samples were then permeabilized in acetone at -20°C or 10pg/ml proteinase K at room temperature, blocked in 10% normal goat serum and incubated with primary antibodies (3A10 antibody at 1:200 dilution, Developmental Studies Hybridoma Bank; HB9 antibody MNR2 at 1:400 dilution, Developmental Studies Hybridoma Bank; phosphorilated-histone 3 Ser10 antibody at 1:200 dilution, Cell Signalling #9701; all antibodies incubated with 5% normal goat serum). After washing, samples were incubated with appropriate secondary antibodies conjugated with AlexaFluor Dyes Al488, Al568 or Al633 (1:2000 dilution; Molecular Probes, Life Technologies).

### Transmission Electron Microscopy

Tissue was prepared for TEM as previously described [20, 48]. Briefly, zebrafish were terminally anaesthetised in tricaine and incubated immediately in primary fixative (4% paraformaldehyde 2% glutaraldehyde in 0.1M sodium cacodylate buffer) with microwave stimulation. Samples were then incubated with secondary fixative (2% osmium tetroxide in 0.1M sodium cacodylate/imidazole buffer) with microwave stimulation; stained en bloc with a saturated uranyl acetate solution with microwave stimulation, and dehydrated in an ethanol series and acetone with microwave stimulation. Samples were then embedded in EMbed-812 resin (Electron Microscopy Sciences), and sectioned using a Reichert Jung Ultracut Microtome. Sections were cut at comparable somite levels by inspection of blocks under a dissection microscope, and stained in uranyl acetate and Sato lead stain. TEM images were taken with a Phillips CM120 Biotwin TEM.

### Light Microscopy and image acquisition

Larvae were anesthetised with tricaine and embedded in 1.3–1.5% low melting point agarose in embryo medium with tricaine. Confocal images and time-lapses were acquired using Zeiss LSM 710 and 780 confocal microscopes; except for OPC specification analysis and Acridine Orange staining analysis which were imaged with a Zeiss Imager Z1 equipped with an Apotome2 structured illumination unit. Images of the anterior region of the spinal cord were acquired by aligning the edge of the visual field to the urogenital opening somite, and the posterior region by aligning the visual field to the last somite in the spinal cord, using a 20X objective (Zeiss Plan-Apochromat 20x dry, NA = 0.8, Carl Zeiss Microscopy), zoom 1x. This resulted in a 425μm long image in the Zeiss LSM710/780 confocal microscope and in a 488μm long image in the Zeiss Imager Z1, corresponding to about 5–6 somites in the 60-96hpf zebrafish spinal cord (out of about 30). In all cases, Z-stacks were acquired of the entire depth of the spinal cord, with a Z-interval of 2 μm or 2.1 μm in the case of time-lapses. For time-lapses, agarose-embedded fish were imaged on a temperature controlled microscope stage at 28.5°C, and z-stacks were acquired at 14min intervals overnight using the Zeiss LSM710/780 confocal microscopes. Larvae were checked for good blood circulation and general health in the morning. All images and movies represent a lateral view of the spinal cord, anterior to the left and dorsal on top.

### Image processing and analysis

Most image processing and analysis was done in Fiji (a distribution of ImageJ). Figure panels were produced using Fiji and Adobe Illustrator CS8. For figures, maximum-intensity projections of Z-stacks were made, and a representative x-y area was cropped. For most images, processing included only global change of brightness and contrast, further processing is as follows.

*TEM analysis*: Electron micrograph tiles were aligned automatically using the automated Photomerge tool in Adobe Photoshop. Axons containing identifiable neurofilament cross-sectional profiles (10nm), with a roughly circular profile and located in the white matter were traced and measured in Adobe Photoshop or Fiji and their area was used to determine the corresponding diameter. Small-diameter axons in medial spinal cord were counted in sample 8.2μm^2^ regions. All tracing was performed while blinded to genotype.

*Cell counts*: Oligodendrocyte number was counted through z-stacks encompassing the entire depth of the spinal cord in Tg(mbp:EGFP) larvae, in which GFP is localised to the cytoplasm of oligodendrocytes, which facilitates the quantification of all oligodendrocyte cell bodies. OPC number was counted in z-stacks of a lateral view of Tg(olig2:GFP; sox10:mRFP) larvae, in which OPCs express both cytoplasmic GFP and membrane-tethered mRFP; both channels were simultaneously visualized while counting. A grid was superimposed on the image and all cell bodies were counted throughout the z-stack using the cell counter plugin in Fiji. An approach based on unbiased stereological methods was used to ensure that each cell was only counted once (consecutive optical sections were acquired to sample each cell more than once, and each cell was counted only in the first optical section it appears). Counts in PH3 and HB9 immunostained larvae were performed while blinded to genotype.

*Time-lapse image analysis*: Z-stacks were maximum intensity-projected for each time point; the olig2:EGFP channel was registered between timepoints using the ‘Register virtual stack slices’ plugin in Fiji, and the ‘Transform virtual stack slices’ plugin was used to apply the same transformations to the sox10:mRFP channel. Fiji’s Cell Counter plugin was used to count OPC mitoses and deaths.

### Statistical analyses

All graph-making and statistical tests were carried out using GraphPad Prism, except for 2-sample Poisson rate tests, used to test the rate of OPC mitoses and oligodendrocyte-lineage cell deaths which were carried out using Minitab. All other data were averaged per biological replicate (N represents number of larvae). Unless otherwise noted, data was assumed to be normally distributed and was compared between WT and mutant groups using a two-tailed unpaired Student’s t-test, considering P<0.05 as a significant result. Additionally, the distribution of all data was also compared between WT and mutant using the non-parametric Mann-Whitney *U* test, which does not require the assumption of normality. All comparisons of distributions in the WT and mutant groups maintained the same significance as using the parametric t-test apart from the rate of OPC mitoses per 12-hour in the posterior spinal cord, where MW test P = 0.093. Throughout the figures, error bars illustrated mean ± standard deviation (SD). Statistical significance is indicated as follows: * P<0.05, ** P<0.01, *** P<0.001.

## Results

### *kbp* mutants lack reticulospinal axons in the posterior spinal cord

**Fig 1:**
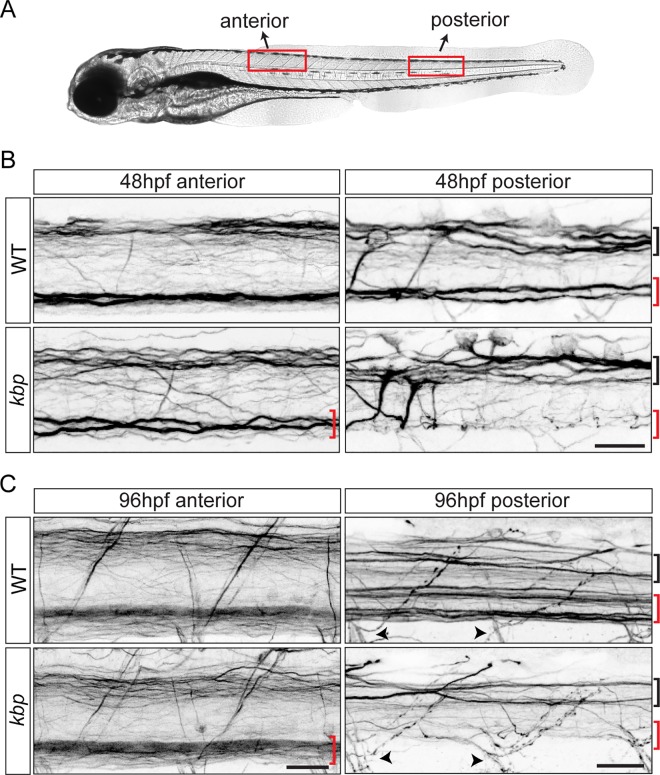
*kbp* mutants lack reticulospinal axons in the ventral spinal cord. *A*, Location of ‘anterior’ and ‘posterior’ regions analysed. *B-C*, 3A10 immunostained spinal cords of 48hpf (B) or 96hpf (C) WT and *kbp* mutant larvae; mutants lack the ventral reticulospinal axons (red bracket), but the dorsal tract (black bracket) and motor axon exit points (arrowhead) appear intact. Scalebars: 25μm.

To test how the amount of target axonal surface regulates oligodendrocyte lineage development, we sought to analyse *kbp* mutants in which reticulospinal axons do not fully grow in length. By immunostaining with 3A10 antibody, which labels a neurofilament-associated epitope, we find that in wildtype siblings (WT) reticulospinal axons have grown along the entire ventral spinal cord by 48 hours postfertilization (hpf) (N = 15/15 larvae), whereas *kbp* mutants lack most reticulospinal axons in the posterior spinal cord (somite levels 20–24, N = 10/10 larvae, Fig 1A and 1B). This is not a transient outgrowth delay, as by 96hpf reticulospinal axons are still absent from the posterior spinal cord of mutants (Fig 1C). In the anterior spinal cord (somite levels 9–13), some reticulospinal axons still appear qualitatively present. Other axons, including those in the dorsal tract and motor axons, appear similar to WT in both the anterior and posterior spinal cord (Fig 1B and 1C).

**Fig 2:**
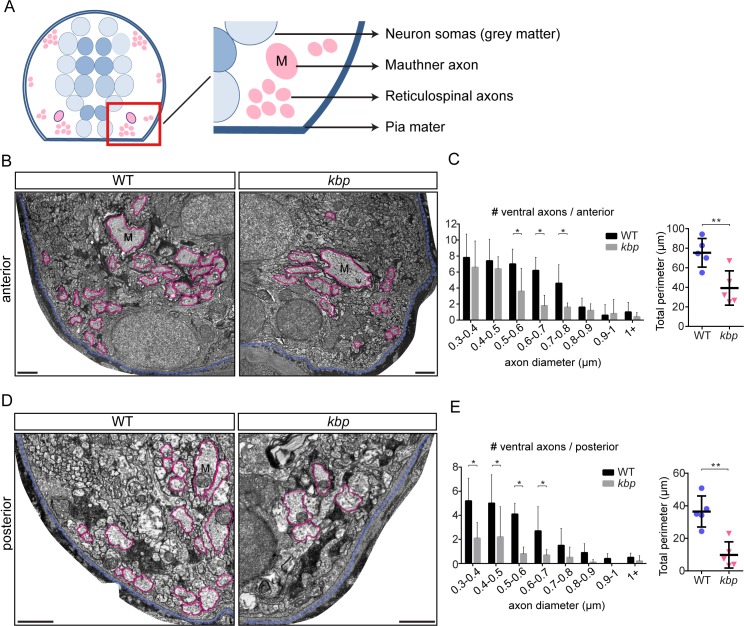
Fewer large-calibre axons in the ventral spinal cord of *kbp* mutants. *A*, Diagram of transversal section of spinal cord. Grey matter is in the centre (blue) and white matter or axonal cross-sectional profiles around it (pink). Red box indicate approximate areas shown in B and D. *B*, *D*, Transmission electron micrographs of ventral regions of the anterior (B) and posterior (D) spinal cord at 72hpf. Large axons are traced in pink, pia mater in blue. M = Mauthner axon. Scale bar: 1μm. *C*, *E*, Distribution of large ventral axons in WT and *kbp* mutant shows significant reductions in the number of large (reticulospinal) axons in mutants in both the anterior (C) and posterior (E) spinal cord. Graphs on right show that the total perimeter belonging to large axons is significantly reduced in mutants in both regions. Data from N = 5 WT and N = 5 mutant larvae. * indicates P<0.05; ** indicates P<0.01; see text for details. Error bars indicate ± SD.

To quantitatively assess the reduction in reticulospinal axons in *kbp* mutants, we examined the ultrastructure of the spinal cord by transmission electron microscopy at 72hpf. In the ventral posterior spinal cord (Fig 2D), as expected from the 3A10 immunostaining, we found a significant reduction in the number of large-calibre axons (20±5 vs 7±4 axons in WT vs mutant, P = 0.002 in t-test, see distribution in Fig 2E). Concomitantly, we found a 73% reduction in the total perimeter of large-calibre axons in the ventral spinal cord of mutants (36.5±9.5μm vs 9.8±8.1μm in WT vs mutant, P = 0.001 in t-test, Fig 2E), a parameter that reflects the axonal surface available for potential interactions with oligodendrocyte-lineage cells. In the anterior spinal cord we found a significant, but comparatively smaller reduction in the number of large ventral axons (36±4 vs 22±9 axons in WT vs mutant, P = 0.016 in t-test, see distribution in Fig 2B), and in their total perimeter (75.4±14.6μm vs 39.2±17.5μm in WT vs mutant, P = 0.008 in t-test, Fig 2C). The average area and perimeter of the remaining large axons in the spinal cord, including the Mauthner axon in the anterior region, is similar to WT (S1 Table), suggesting that genetic disruption of *kbp* affects the outgrowth and length of reticulospinal axons, but not their calibre.

**Fig 3:**
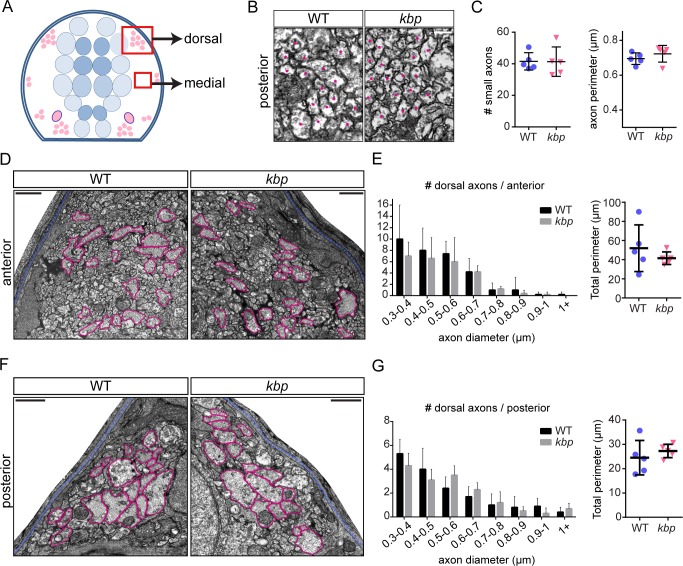
Dorsal and medial axons are not affected in *kbp* mutants. *A*, Diagram of hemi-spinal cord transversal section with red boxes indicating dorsal and medial regions shown in B, D and F. *B*, Transmission electron micrographs of medial region of the posterior spinal cord, with dots indicating small-diameter axons. Scale bar: 0.2μm. *C*, number and average perimeter of medial axons. *D*, *F*, Electron micrographs of dorsal region of the anterior (D) and posterior (F) spinal cord. Large axons are traced in pink, pia mater in blue. Scale bar: 1μm. *E*, *G*, Similar distributions of large dorsal axons in WT and *kbp* mutant anterior (E) and posterior (G) spinal cord. The total perimeter belonging to large axons is similar in WT and mutants. Data from N = 5 WT and N = 5 mutant larvae. Error bars indicate ± SD.

Remarkably, the number and distribution of large axons in the dorsal spinal cord (Fig 3), which are not reticulospinal axons, was similar to WT in both the anterior (32±10 vs 26±2 axons in WT vs mutant, P = 0.200 in t-test, see distribution in Fig 3E) and posterior spinal cord (17±2 vs 16±2 axons in WT vs mutant, P = 0.585 in t-test, see distribution in Fig 3G). The total perimeter was also similar between WT and mutants in both the anterior (52.0±24.4μm vs 42.0±6.6μm in WT vs mutant, P = 0.384 in t-test, Fig 3E) and posterior spinal cord (24.5±7.1μm vs 27.3±2.7μm in WT vs mutant, P = 0.441 in t-test, Fig 3G). The medial region of the spinal cord is normally occupied by many smaller-diameter (<0.5 μm diameter) axons (Fig 3B and 3C), and we found that both the number of such axons and their average perimeter were normal in the mutant posterior spinal cord (42±5 WT vs 41±9 mutant axons in a sample 8.2μm^2^ area, P = 0.968 in t-test; 0.69±0.03 μm WT vs 0.72±0.05μm average perimeter in mutants, P = 0.317 in t-test, Fig 3C).

Therefore, *kbp* mutants have a significant reduction in reticulospinal axonal surface, the first target for myelination by oligodendrocytes in the ventral spinal cord. This reduction ranges from 48% in the anterior spinal cord to 73% in the posterior spinal cord.

### Oligodendrocyte number is reduced in the posterior, but not anterior mutant spinal cord

**Fig 4:**
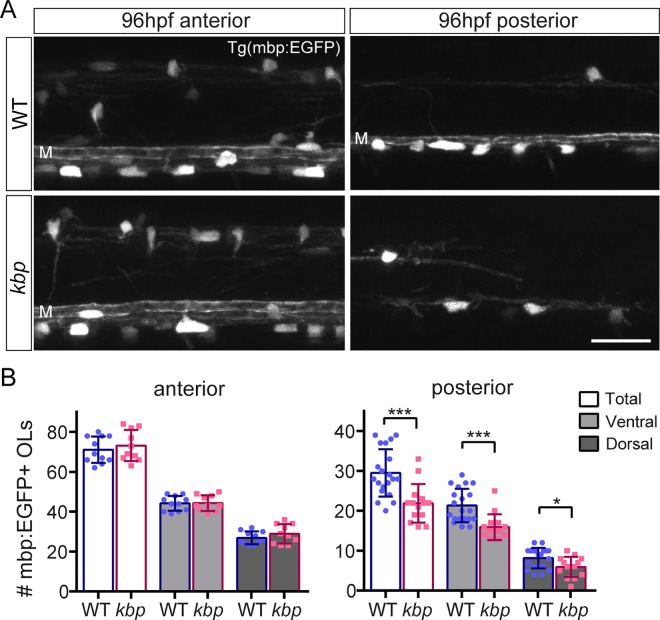
Fewer oligodendrocytes mature in the posterior but not anterior spinal cord of *kbp* mutants. *A*, Spinal cord of 96hpf Tg(mbp:EGFP) larvae with mature oligodendrocyte somas labelled. The myelin sheath of the very large diameter Mauthner axon (M), a reticulospinal axon, is visible in the ventral spinal cord except in the posterior region of mutants. Scale bar: 25 μm. *B*, The number of total, ventral and dorsal oligodendrocytes (OLs) is reduced in a 425μm long region of the posterior but not anterior spinal cord in mutants, compared to WTs. Data from N = 11 WT and N = 10 mutants (anterior) and N = 20 WT and N = 14 mutants (posterior). * indicates P<0.05; *** indicates P<0.001; see text for details. Error bars indicate ± SD.

We then sought to determine whether the oligodendrocyte cell population was affected by the reduction in reticulospinal axons. Oligodendrocyte precursor cells differentiate into myelinating oligodendrocytes starting at 60hpf in the zebrafish spinal cord [20]. We counted oligodendrocyte number at 96hpf using our previously established transgenic reporter line Tg(mbp:EGFP), in which EGFP is expressed in myelinating glia under the control of a fragment of the *myelin basic protein* (*mbp*) promoter [20]. We found that the total number of oligodendrocytes was significantly decreased in the posterior spinal cord (30±6 vs 22±5 oligodendrocytes in WT vs mutant; P = 0.0004 in t-test, Fig 4B). In contrast, oligodendrocyte number was unaffected in the anterior spinal cord of *kbp* mutants, despite the nearly 50% reduction in reticulospinal axon surface (71±7 vs 73±8 oligodendrocytes in WT vs mutant, P = 0.510 in t-test, Fig 4B). In the posterior spinal cord, oligodendrocytes in dorsal and ventral regions were reduced (ventral: 21±4 vs 16±3 oligodendrocytes in WT vs mutant, P = 0.0003 in t-test; dorsal: 8±3 vs 6±3 oligodendrocytes in WT vs mutant, P = 0.018 in t-test), despite the normal number of axons in the dorsal spinal cord.

Despite the global disruption to *kbp*, oligodendrocyte number is only reduced in the posterior spinal cord, where reticulospinal axonal surface is also drastically reduced, suggesting that the decreased target axonal surface, rather than *kbp* loss of function, impairs some aspect of oligodendrocyte development. Remarkably, the 73% reduction in reticulospinal axonal surface in the posterior spinal cord of mutants is accompanied by a comparatively smaller 27% reduction in oligodendrocyte number. Thus, the development of the majority of the oligodendrocyte population does not absolutely require normal levels of target axonal surface, suggesting that target axons may exert a regulatory, but not essential, role over the oligodendrocyte lineage in vivo.

We then sought to investigate which aspects of oligodendrocyte development were affected in the posterior spinal cord that led to the reduction in cell number by 96hpf.

### OPCs are specified independently of reticulospinal axons

**Fig 5:**
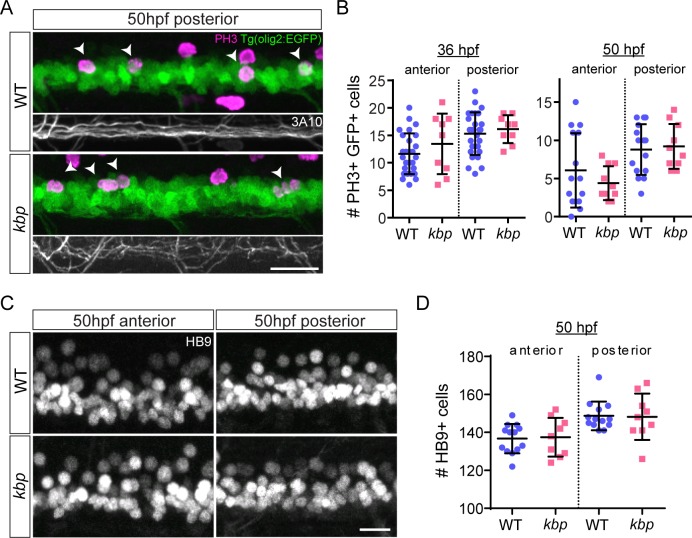
pMN progenitor proliferation and motor neuron generation are unaffected by reduction in reticulospinal axons. *A*, phospho-Histone 3 immunostained 50hpf Tg(olig2:EGFP) larvae. EGFP+ PH3+ cells indicated by arrowheads; 3A10 panels show labelled reticulospinal axons, or their absence, in corresponding area. Scale bar: 20μm. *B*, similar number of PH3+ EGFP+ cells between WT and mutants in 425μm long regions of the spinal cord 36–50 hpf. Data from N = 27 WT and N = 9 mutants (36hpf) and N = 15 WT and N = 10 mutants (50hpf). *C*, HB9 immunostained 50hpf larvae to label motor neurons. Scale bar: 12.5μm. *D*, similar number of motor neurons between WT and mutant in 150μm long regions of the spinal cord. Data from N = 13 WT and N = 9 mutants. Error bars indicate ± SD.

OPCs are born in the pMN domain of the ventral spinal cord [49, 50], where olig2+ progenitor cells first generate hb9+ motor neurons [51, 52], and later switch to producing olig2+ sox10+ OPCs. To determine whether reticulospinal axons regulate the pMN cell population, we quantified the number of mitotic (phosphorylated histone 3-positive) olig2+ pMN progenitors in the posterior spinal cord of *kbp* mutants crossed into the Tg(olig2:EGFP) transgenic line (Fig 5A and 5B). At 36 hpf, when OPCs are first specified [44], the number of mitotic olig2:EGFP+ cells was similar between WT and mutants in both the anterior (12±4 vs 13±6 cells in WT vs mutants, P = 0.270 in t-test) and posterior spinal cord (15±4 vs 16±3 cells in WT vs mutants, P = 0.560 in t-test, Fig 5B). Later on at 50hpf (Fig 5A and 5B), when most motor neurons have terminally differentiated [53], the number of mitotic pMN progenitors remained similar in the anterior (6±5 vs 4±2 cells in WT vs mutants, P = 0.324 in t-test) and posterior spinal cord (9±3 vs 9±3 cells in WT vs mutants, P = 0.761 in t-test, Fig 5B). Additionally, the number of postmitotic HB9+ motor neurons was similar between WT and mutants in the anterior (137±8 vs 137±10 cells in WT vs mutants, P = 0.845 in t-test, Fig 5C and 5D) and posterior spinal cord (149±8 vs 148±12 cells in WT vs mutants, P = 0.912 in t-test, Fig 5C and 5D). Thus, neither *kbp* nor reticulospinal axons seem to regulate the proliferation of pMN progenitors or the generation of motor neurons.

**Fig 6:**
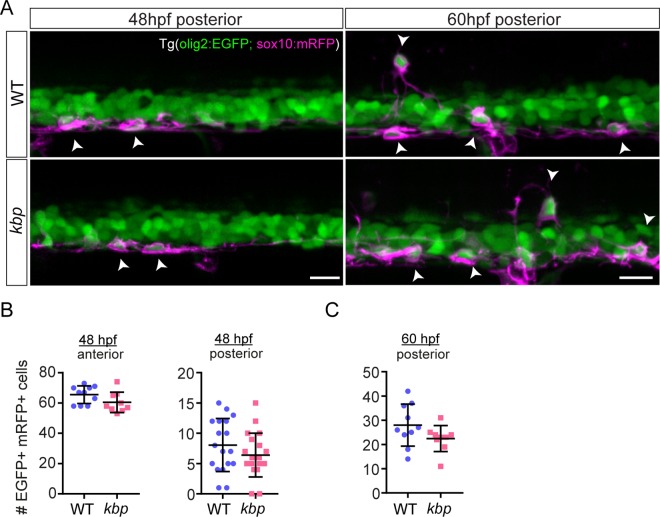
OPCs are specified normally despite reduction in reticulospinal neurons. *A*, Posterior region of 48-60hpf Tg(olig2:EGFP; sox10:mRFP). EGFP+ mRFP+ cells (OPCs) indicated by arrowheads. At 60hpf some OPCs have migrated dorsally. Scale bar: 20 μm. *B-C*, Similar number of OPCs in 448μm-long regions of the spinal cord between WT and mutants at 48hpf (B) and 60hpf (C). Data from N = 10 WT and N = 9 mutants (48hpf anterior and 60hpf posterior) and N = 18 WT and N = 20 mutants. Error bars indicate ± SD.

We then investigated the appearance of oligodendrocyte precursor cells (OPCs), which can be visualised using the double transgenic reporter Tg(olig2:EGFP; sox10:mRFP) [54] (Fig 6A). In this reporter, cytoplasmic EGFP and membrane-tethered RFP are only expressed simultaneously in OPCs in the spinal cord. At 48hpf, the anterior region has OPCs distributed between the ventral and dorsal spinal cord, and, as expected, their number is similar between WT and mutants (66±6 vs 60±7 OPCs in WT vs mutant, P = 0.097 in t-test, Fig 6B). In the posterior region OPC specification has just begun and a similar number of OPCs is also present in WT and mutants (8±4 vs 6±4 OPCs in WT vs mutant, P = 0.209 in t-test, Fig 6A and 6B). Later on at 60hpf the number of OPCs remains similar in the posterior spinal cord (28±9 vs 22±5 OPCs, P = 0.116 in t-test, Fig 6C), indicating that the initial specification of OPCs is not affected by disruption to *kbp*, or the reduction in reticulospinal axons.

### OPCs migrate independently of reticulospinal axons

Having determined that the initial appearance of OPCs was normal in the posterior spinal cord of *kbp* mutants lacking most reticulospinal axons, we then wondered how the reduction in mature oligodendrocytes two days later could come about. We reasoned that the severe reduction in reticulospinal axonal surface could cause abnormal migration, proliferation, differentiation or survival of oligodendrocyte-lineage cells. To distinguish between these effects, we took advantage of the amenability of zebrafish for live imaging and performed time-lapse analysis of the double transgenic reporter Tg(olig2:EGFP; sox10:mRFP), in which the membrane-tethered mRFP reporter allows us to monitor the location, morphology and behaviour of oligodendrocyte-lineage cells and their processes. We started these analyses at around 60hpf and imaged over a period of 11±3 hours in the anterior spinal cord or 12±3 hours posterior spinal cord (see representative movies of the posterior spinal cord of a WT in S1 Movie and of a *kbp* mutant in S2 Movie).

**Fig 7:**
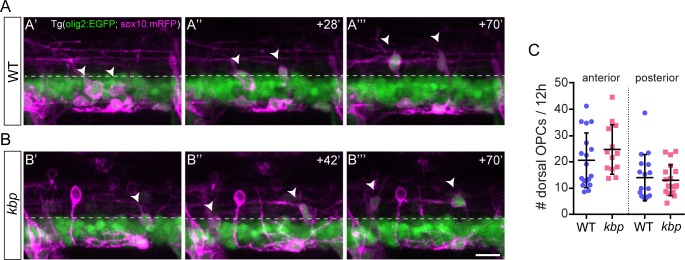
OPCs migrate dorsally normally despite reduction in reticulospinal axons. *A*, *B*, Time-lapse stills of Tg(olig2:EGFP; sox10:mRFP), WT (A) or *kbp* mutant (B) larvae, showing two OPCs (arrowheads) migrating dorsally above the olig2:EGFP band of motor neurons and pMN precursors (dashed line). Scale bar: 10μm. *C*, The rate at which OPCs accumulate in the dorsal spinal cord is similar between WTs and mutants in 425μm long regions. Data from N = 18 WT and N = 13 mutants (anterior) and N = 17 WT and N = 19 mutants (posterior). Error bars indicate ± SD.

Shortly after their specification in the ventral spinal cord, a subset of OPCs migrates towards the dorsal spinal cord (Fig 6A, 60hpf posterior). It is thought that OPC-OPC interactions contribute to the distribution and tiling of OPCs [44], but the role of axons in regulating OPC migration in the zebrafish spinal cord has not been assessed. We wondered whether the severe reduction of ventral reticulospinal axons might dysregulate the dorsal migration of OPCs. We analyzed the dynamics of OPC migration starting at 60hpf. OPCs migrate dorsally above the olig2:EGFP+ band in the ventral spinal cord in WT and mutant larvae (Fig 7A and 7B), and with similar rates in the anterior (21±10 vs 25±9 OPCs/12-hour in WT vs mutant, P = 0.265 in t-test, Fig 7C) and posterior spinal cord (14±9 vs 13±6 OPCs/12-hour in WT vs mutant, P = 0.671 in t-test, Fig 7C). These results indicate that neither *kbp* nor reticulospinal axons regulate the migration of OPCs into the dorsal spinal cord. Furthermore, these analysis showed that aberrant patterns of migration do not contribute to the dearth of oligodendrocytes in the posterior spinal cord of *kbp* mutants.

### OPCs proliferate less in *kbp* mutants

**Fig 8:**
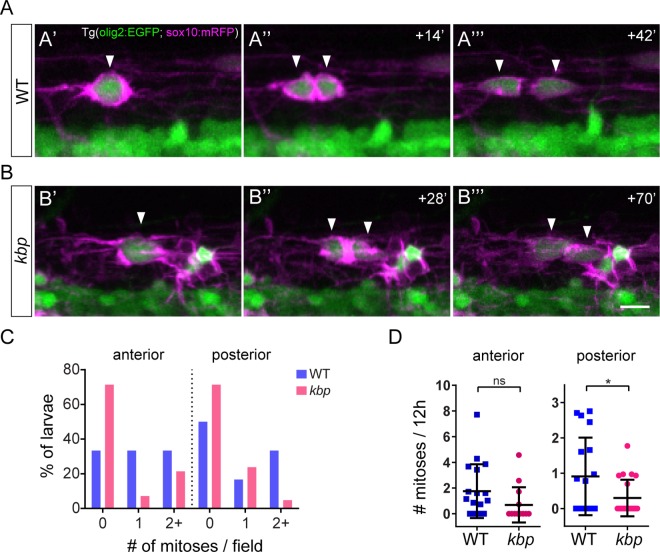
Impaired OPC proliferation in the posterior spinal cord of *kbp* mutants. *A*, *B*, Time-lapse stills showing representative OPC mitoses in WT (A) and *kbp* mutants (B); arrowheads indicate mother and daughter cells. Scale bar: 10 μm. *C*, Proportion of larvae with OPC mitoses. Fewer mutants have any OPC mitoses compared to WTs. *D*, Rate of OPC mitoses (per 12-hour) is significantly decreased only in the posterior spinal cord of *kbp* mutants. Data from N = 18 WT and N = 14 mutants (anterior) and N = 18WT and N = 21 mutants (posterior). * indicates P<0.05; see text for details. Error bars indicate ± SD.

To test whether reticulospinal axons might influence OPC proliferation, we assessed the frequency of OPC mitoses in our time-lapse analyses. We identified OPC mitoses in both WT and mutants: the processes of an OPC partially retracted; mRFP+ membrane accumulated in the centre of the cell, resembling a cleavage furrow, and the cell’s EGFP+ cytoplasm divided (Fig 8A and 8B). We found that a greater proportion of the mutant larvae analysed had zero OPC mitoses compared to WT (Fig 8C). In the posterior spinal cord, the rate of occurrence of OPC mitoses was significantly reduced in mutants (0.9±1.1 vs 0.3±0.5 mitoses/12-hour in WT vs mutants, P = 0.029 in t-test, Fig 8D), while in the anterior spinal cord a similar trend did not reach statistical significance (1.8±2 vs 0.7±1.4 mitoses/12-hour in WT vs mutants, P = 0.107 in t-test, Fig 8E). We additionally employed a statistical analysis that takes into account the duration of each larva’s time-lapse imaging and the total number of events in all larvae (S2 Table). Using a two-sample Poisson rate exact test, the rate of occurrence of OPC mitoses also shows a significant reduction in the posterior (7.2∙10^−2^ mitoses/12-hour in 176h imaged in N = 18 WT vs 2.1∙10^−2^ mitoses/12-hour in 218h from N = 21 mutants; P = 0.004), but not anterior spinal cord (11.5∙10^−2^ mitoses/12-hour in 197h imaged from N = 18 WT vs 6.6∙10^−2^ mitoses/12-hour in 130h imaged from N = 14 mutants; P = 0.153). Interestingly, OPC mitoses are reduced in the ventral subpopulation of OPCs, where fewer reticulospinal axons are present, but also in the dorsal subpopulation of OPCs (S2 Table).

Thus, we observe a low level of OPC proliferation in the wildtype zebrafish spinal cord at the stages immediately following OPC specification. In the posterior spinal cord of *kbp* mutants, where reticulospinal axonal surface is drastically decreased, the low level of OPC proliferation is further reduced, suggesting that there is a dearth of proliferative signals normally provided by reticulospinal axons.

### A subset of OPCs dies in the absence of reticulospinal axons

**Fig 9:**
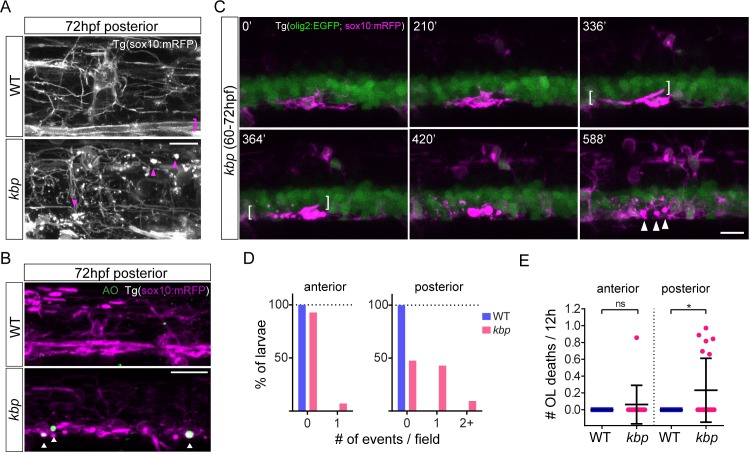
Impaired survival of a subset of oligodendrocyte-lineage cells in the posterior spinal cord of *kbp* mutants. *A*, High-magnification live-imaging of the posterior spinal cord of 72hpf Tg(sox10:mRFP) larvae, with Mauthner axon myelin sheaths in WTs (pink bracket) and mRFP+ debris in mutants (pink arrowheads). Scale bars: 10 μm *B*, Posterior spinal cord of 72hpf Tg(sox10:mRFP) larvae stained with Acridine Orange; mutants have AO+ foci (arrowheads). See text for details. Scale bar: 20 μm. *C*, Time-lapse stills showing a mutant oligodendrocyte forming a myelin sheath (brackets) and then dying, originating mRFP+ debris (arrowheads). Scale bar: 10μm. *D*, Proportion of larvae with oligodendrocyte death events: essentially only mutants show any events, only in the posterior spinal cord. *E*, Rate of oligodendrocyte (OL) death (per 12-hour) is significantly elevated only in the posterior spinal cord of *kbp* mutants. Data from N = 18 WT and N = 14 mutants (anterior) and N = 18WT and N = 21 mutants (posterior). * indicates P<0.05; see text for details. Error bars indicate ± SD.

The reduction in an already low level of OPC proliferation by 72hpf does not appear to reflect the magnitude of the effect on oligodendrocyte number by 96hpf. Therefore, we wondered whether the survival of oligodendrocyte-lineage cells could also be impaired. Using the Tg(sox10:mRFP) reporter line allowed us to monitor the integrity of oligodendrocyte-lineage cells. In the posterior spinal cord of mutants we frequently observed bright clusters of mRFP+ fluorescence starting around 72hpf (in 37/47 mutants vs 3/54 WT, Fig 9A). Such mRFP+ debris was not present in the mutants’ anterior spinal cord, or in the WT spinal cord. We applied a brief pulse of Acridine Orange, which facilitates visualization of dying cells [55, 56], to 72hpf Tg(sox10:mRFP) larvae (Fig 9B) and found a significant increase in AO+ foci surrounded by mRFP+ debris in the posterior (0.1±0.3 foci in N = 20 WT vs 1.6±1 foci in N = 17 mutants, p<0.0001 in t-test) but not in the anterior region of mutants, compared to WT (0.8±1 foci in N = 20 WT vs 1.2±1 foci in N = 17 mutants, P = 0.279 in t-test). We then investigated the origin of debris in our time-lapse studies between 60-72hpf. In the posterior spinal cord of mutants, we identified individual oligodendrocyte-lineage cells whose processes disintegrated, generating debris, followed by the disappearance of the olig2:EGFP+ cytoplasm (Fig 9C, S3 Movie). Although in individual mutant larvae such occurrences were infrequent (1 event in 9/21 mutants, 2 events in 2/21 mutants, Fig 9D), they never occurred in posterior spinal cord of the 18 WT imaged, and did not occur in the anterior spinal cord, in WT or mutant animals (Fig 9D and 9E). The rate of oligodendrocyte-lineage cell deaths in individual larvae was significantly elevated in the posterior spinal cord of mutants (0±0 vs 0.23±0.38 cell deaths/12-hour in WT vs mutants, P = 0.014 in t-test, Fig 9E), but not in the anterior spinal cord (0±0 vs 0.06±0.22 cell deaths/12-hour in WT vs mutants, P = 0.264 in t-test, Fig 9E). Employing the two-sample Poisson rate exact test which takes into account the total imaged time (S2 Table), similarly resulted in a significant increase in the rate of oligodendrocyte-lineage cell death in the posterior spinal cord (0 vs 3.4∙10^−2^ cell deaths/12-hour in WT vs mutants; P = 0.002), but not in the anterior spinal cord (0 vs 0.7∙10^−2^ cell deaths/12-hour in WT vs mutants; P = 0.678). Furthermore, essentially all oligodendrocyte-lineage cell deaths occurred in the ventral spinal cord (S2 Table), where reticulospinal axons are absent, suggesting that the missing reticulospinal axons provide a local survival cue to oligodendrocytes.

Collectively, these data indicate that a subset of oligodendrocyte-lineage cells die in the posterior spinal cord of *kbp* mutants, where there is a drastic reduction of reticulospinal axonal surface, contributing to a reduction in the number of mature oligodendrocytes at 96hpf. Given that oligodendrocyte-lineage cells in the anterior spinal cord of mutants survive normally, despite also lacking *kbp* function, impaired oligodendrocyte survival in the posterior spinal cord is more likely due to the reduction in reticulospinal axons in this region than to the global *kbp* disruption. Thus, the first axons fated for myelination, reticulospinal axons, support the survival of oligodendrocyte-lineage cells in the developing zebrafish spinal cord.

### Oligodendrocytes differentiate on schedule in the absence of reticulospinal axons

Despite the effects of a 73% reticulospinal axon reduction on proliferation and survival, the majority of oligodendrocytes (also 73%) differentiate by 96hpf, suggesting that target axons are not absolutely required for oligodendrocyte development. We wondered, however, whether individual OPCs took longer to differentiate in the near absence of their target axons, or whether the timing of their differentiation is instead independent of axons as it seems to be in vitro [21,27–29].

**Fig 10:**
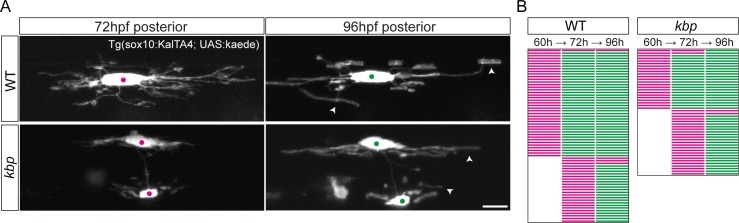
Oligodendrocytes differentiate on time in the posterior spinal cord of *kbp* mutants. *A*, Time-course of sparsely labelled oligodendrocyte-lineage cells in Tg(sox10:KalTA4; UAS:kaede) larvae between 72-96hpf in the posterior spinal cord, showing myelin sheath formation by 96hpf (arrowheads). Scale bar: 10 μm. *B*, Time course profiles of 66 WT and 48 mutant cells; magenta indicates OPC stage, green indicates oligodendrocyte. Most cells mature by 96hpf in both WT and mutants.

To investigate the timing of oligodendrocyte differentiation in the posterior spinal cord of *kbp* mutants, we took advantage of our ability to perform time-course studies of the same individual cells over time, while imaging the processes and myelin sheaths of individual oligodendrocytes at single-cell resolution. We wanted to track the fate of individual OPCs at three time-points: 48hpf, prior to myelination, and 60 and 72hpf, when myelination ensues. To do this, we sparsely labelled oligodendrocyte-lineage cells by crossing the Tg(sox10:KalTA4) driver line [45] with a reporter Tg(UAS:Kaede) [46]. In the double Tg(sox10:KalTA4; UAS:kaede) line, sox10-expressing cells such as OPCs produce the transcription factor KalTA4, which in turn binds to the Upstream Activating Sequence (UAS) in the reporter line and induces expression of the fluorescent protein Kaede. Due to inheritable epigenetic silencing of the repetitive UAS sequences [57–59], expression of UAS-driven reporters is often sparse, allowing the identification of isolated cells and their individual morphology. In the posterior spinal cord, we identified individual Kaede+ OPCs with numerous ramified processes at 60hpf and 72hpf, and analysed whether by 96hpf they had differentiated into myelinating oligodendrocytes elaborating tubular, long myelin sheaths at the end of their processes (Fig 10). In the posterior spinal cord of both WT and mutants, the vast majority of identified OPCs differentiated into myelinating oligodendrocytes by 96hpf (Fig 10B; 62/66 WT OPCs, 46/48 mutant OPCs; P = 1 in two-tailed Fisher’s test). This result indicates that even in a drastically altered axonal environment, OPCs differentiate into oligodendrocytes in a timely manner, suggesting that the timing for oligodendrocyte differentiation in vivo is independent both of *kbp* and of the axons targeted for myelination.

## Discussion

Here, we characterized *kbp* mutant zebrafish larvae where the number of large diameter reticulospinal axons normally fated for myelination in the spinal cord is reduced. This allowed us to investigate how target axons regulate oligodendrocyte development in vivo. We show that the initial development of oligodendrocytes does not require their axon targets or *kbp*, but that later aspects of OPC proliferation and oligodendrocyte survival are somewhat affected in a reticulospinal axon-dose-dependent manner: essentially only in the posterior spinal cord of mutants, where most reticulospinal axons are absent (S1 Fig). Remarkably, in the anterior spinal cord of *kbp* mutants, the oligodendrocyte lineage cell population develops normally, despite a nearly halved target axonal surface, indicating that a normal number of target axons is not strictly necessary for oligodendrocyte development. In the posterior spinal cord of mutants, where only around a quarter of target axonal surface remains, oligodendrocyte lineage cell proliferation and development are somewhat affected; but the majority of oligodendrocytes (73%) still develops by 96hpf. Importantly, oligodendrocyte-lineage cells in the anterior spinal cord also carry the *kbp* mutation, but remain unaffected–indicating that this phenotype is unlikely to be due to a cell-autonomous effect of the *kbp* mutation in oligodendrocytes. In the future, however, cell type-specific conditional targeting of *kbp* in oligodendrocytes, e.g. using the Cre-lox system, would more specifically test its possible role(s) in oligodendrocyte development and myelination. Conditional targeting approaches could also be used to test the possible influence of *kbp* in additional cell types of the nervous system in regulating oligodendrocyte development and myelination. However, our data showing normal development of the oligodendrocyte lineage in the anterior spinal cord suggests that the phenotypes observed in the posterior spinal cord of *kbp* mutants are more likely due to the accompanying proportionately larger decrease in target axons in the posterior region.

### Target axons do not regulate the initial stages of oligodendrocyte development

We found that the early stages of OPC specification and migration were not affected by the disruption of *kbp* function or of reticulospinal axons, supporting a previous study where neonatal transection of the retina, which causes all optic nerve axons to degenerate, did not prevent the appearance of NG2+ OPCs or affect their distribution [10]. We found that, unlike neighbouring dopaminergic projection axons [53], reticulospinal axons (and *kbp*) did not regulate pMN progenitor proliferation, motor neuron number or the appearance of the first OPCs, suggesting that OPC specification is independent of target axon-derived signals. Following specification, a subset of OPCs migrates dorsally in the zebrafish spinal cord [44]. OPC-OPC interactions regulate OPC distribution prior to myelination, but the molecular mechanisms that induce the dorsal migration of OPCs in the zebrafish spinal cord are unknown. Our model allowed us to investigate whether reticulospinal axons provide a signal that promotes dorsal OPC migration. We found that the number of dorsal OPCs and their rate of migration are independent of reticulospinal axons, and of *kbp* function. The cue to initiate dorsal migration may be provided by other cells or structures, such as blood vessels [60], or it may simply depend on OPC density in the ventral spinal cord. In fact, *fbxw7* mutants, in which the pMN domain generates an excess of OPCs, have more dorsal OPCs than normal [61]; whereas *pescadillo* mutants [62] or *swap70* morphants [63], which generate fewer total OPCs, have fewer dorsal cells. Combined with our results, this suggests that the initiation of dorsal migration is, at least in part, dependent on OPC-OPC interactions and independent of reticulospinal axons.

### Target axons promote OPC proliferation and oligodendrocyte survival

During and after migration, OPCs proliferate to populate the nervous system, before differentiating into myelinating oligodendrocytes. An early study showed that neonatal transection of the optic nerve did not affect OPC proliferation [10]; whereas recent studies showed that optogenetic stimulation of the premotor cortex can induce OPC proliferation [17], suggesting potential regional differences in axonal regulation of OPC proliferation. In the optic nerve, transection during active myelination causes oligodendrocytes to die [10, 12, 13], indicating that axons deliver a survival signal to newly-differentiated oligodendrocytes. Our time-lapse analyses of combined membrane-tethered and cytoplasmic fluorescent reporters allowed us to directly and specifically detect OPC mitoses and oligodendrocyte-lineage cell death in the spinal cord. We found that individual WT larvae have few OPC mitoses and essentially no oligodendrocyte deaths between 60-72hpf, suggesting that most OPCs in the early zebrafish spinal cord are produced by specification from pMN domain progenitors. Interestingly, in the much larger mammalian developing CNS, there is an initial over-production of OPCs and oligodendrocytes [64, 65]. Many newly differentiated oligodendrocytes die in the mammalian CNS, and all that survive are associated with axons [13, 66], suggesting that competition for limited amounts of a survival signal matches oligodendrocytes to axonal surface [67].

In *kbp* mutants, especially in the posterior spinal cord which lacks most reticulospinal axons, we found OPC mitoses to be even less frequent than in WT, suggesting that axons do promote OPC proliferation to a certain extent in the early developing zebrafish spinal cord. Strikingly, we also find that a subset of ventral oligodendrocyte-lineage cells dies specifically in the posterior spinal cord of mutants, indicating that reticulospinal axons promote oligodendrocyte survival in zebrafish. Interestingly, whereas OPC mitoses were reduced both in the ventral and dorsal spinal cord, oligodendrocyte-lineage cell death events occurred only in the ventral spinal cord. This suggests that the survival signal normally provided by the missing reticulospinal axons may depend on contact between axons and oligodendrocytes, whereas the proliferative cue may act at a distance, perhaps as a secreted mitogen. In fact, secreted Platelet-derived growth factor is the classical mitogen for OPCs [68–70], and recent work identified Neuroligin-3 as a neuronal activity-dependent secreted mitogen for OPCs [16]. Although the scale of OPC proliferation in the early zebrafish CNS is quite different from that in the mammalian CNS, it would be interesting to know whether its molecular regulation is conserved. Given our data that very few OPCs proliferate normally in the wildtype spinal cord, studying proliferation in a different region of the CNS or at a later stage may provide a better platform in which to address this question.

Collectively, our data suggest that axonal regulation of OPC proliferation and oligodendrocyte survival may be conserved in principle, but perhaps not scale, in zebrafish larvae. It will also be interesting to determine the rates of OPC proliferation and oligodendrocyte death at later stages of CNS development in zebrafish, when the tissue volume grows considerably, to address whether the principle of overproduction of cell number and subsequent pruning are also conserved between fish and mammals.

### Target axons do not regulate the timing of oligodendrocytes differentiation

The ventral axonal surface belonging to reticulospinal axons, the initial targets for myelination, was reduced to different levels in the anterior versus the posterior region of the *kbp* mutant spinal cord. Remarkably, normal numbers of oligodendrocytes formed by 96hpf in the anterior spinal cord of mutants despite a reduction of the ventral axonal surface to 52% of WT levels. It will be interesting to determine how the myelinating potential of such a surplus of oligodendrocytes adapts to the altered axonal environment; we are currently investigating the later myelinating behaviour of oligodendrocytes in the *kbp* mutant spinal cord. In the posterior spinal cord, ventral axonal surface was more severely reduced to 27% of WT levels. Interestingly, the majority of oligodendrocytes (73%) still differentiated by 96hpf. We sparsely labelled and tracked individual oligodendrocyte-lineage cells over time to determine whether the drastically altered axonal environment influenced the timing of differentiation of the remaining oligodendrocytes. We found that the majority of oligodendrocytes elaborate myelin sheaths at the normal time in the absence of reticulospinal axons, supporting a previous study in the optic nerve whereby Mbp+ oligodendrocytes appear on time following neonatal transection [10]. Early in vitro studies suggested that an intrinsic timer mechanism controlled the number of OPC divisions and the timing of differentiation [71, 72], but in the complex milieu of a living organism, a variety of signals, not solely neuronal, may also influence such an intrinsic differentiation program. For instance, local physical constraints can regulate OPC differentiation co-cultured with neurons [37]. Intra-lineage signals may regulate the timing of OPC differentiation, whereby OPCs signal to oligodendrocytes and vice-versa to promote their differentiation and maturation [73]. OPCs can regulate the transition to differentiation by secreting factors that regulate angiogenesis, which in turn can affect oligodendrocyte differentiation [74]. Neuronal activity has been shown to promote oligodendrocyte differentiation via secreted signals in vivo: for instance, social experience strongly influences myelination in the mouse pre-frontal cortex [75, 76], and in our own studies we have found that manipulation of neuronal vesicle release can also modulate myelination [18, 77]. In the posterior spinal cord of *kbp* mutants, despite reticulospinal axons being absent, the remaining axons and other structures may be sufficient to promote timely oligodendrocyte differentiation. Such cues promote an internal differentiation programme driven by transcription factors such as Olig1/2, Sox10 and MyRF [78, 79]. These transcription factors upregulate the expression of myelin structural genes, and the cell must then coordinate the synthesis and delivery of myelin components to processes, which will begin the exquisite concentric wrapping and membrane transformation whose details are beginning to be elucidated [80].

## Supporting Information

S1 Fig Graphic summary. (TIF)

S1 Movie Time-lapse of the posterior spinal cord of WT Tg(olig2:EGFP; sox10:mRFP). olig2:EGFP channel in green, sox10:mRFP channel in white. Note dorsal migration of OPCs, OPC proliferation and oligodendrocyte differentiation and myelination of reticulospinal axons in the ventral spinal cord.
(AVI)

S2 Movie Time-lapse of the posterior spinal cord of *kbp*^st23^; Tg(olig2:EGFP; sox10:mRFP). olig2:EGFP channel in green, sox10:mRFP channel in white. Note dorsal migration of OPCs, oligodendrocyte death, and oligodendrocyte differentiation but no myelination of reticulospinal axons in the ventral spinal cord.
(AVI)

S3 Movie Oligodendrocyte-lineage cell death in *kbp*^st23^; Tg(olig2:EGFP; sox10:mRFP). Two examples of oligodendrocyte death in *kbp* mutants, olig2:EGFP channel in green, sox10:mRFP channel in magenta.
(AVI)

S1 Table Transmission Electron Micrograph analysis. (DOCX)

S2 Table Time-lapse analysis–total number of events. (DOCX)
