# Supplementary figures and images for: Oligodendrocyte Development in the Absence of Their Target Axons In Vivo
Source: PLoS One. 2016 Oct 7;11(10):e0164432. doi: 10.1371/journal.pone.0164432 (PMC5055324; doi:10.1371/journal.pone.0164432)

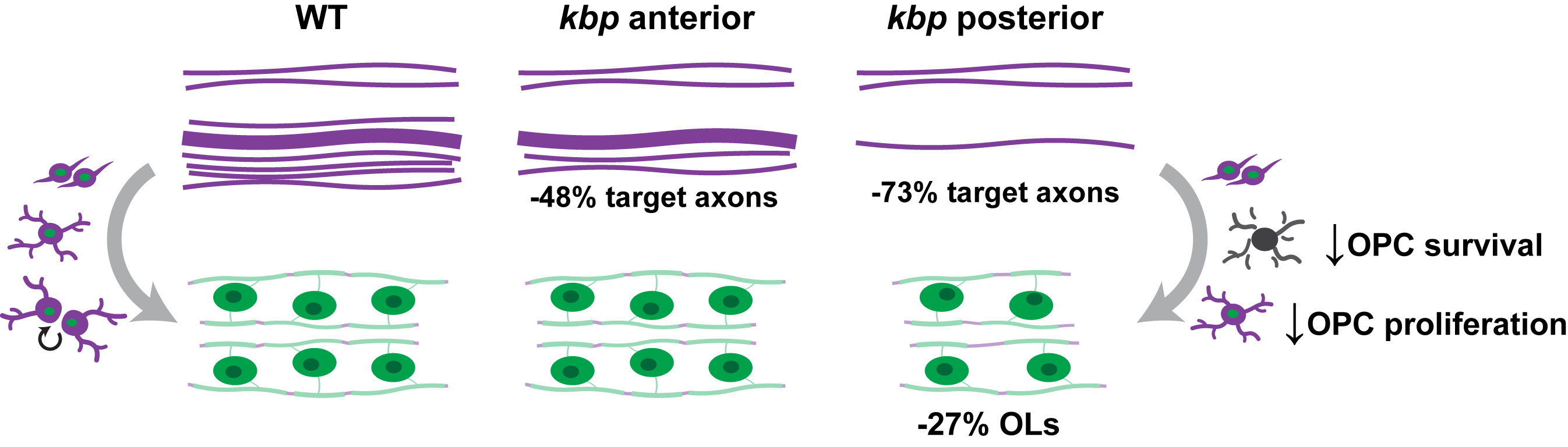

Supplement: S1 Fig — (TIF) [file pone.0164432.s001.tif]
