# Supplementary material for: Oligodendrocyte Development in the Absence of Their Target Axons In Vivo
Source: PLoS One. 2016 Oct 7;11(10):e0164432. doi: 10.1371/journal.pone.0164432 (PMC5055324; doi:10.1371/journal.pone.0164432)
Supplement: S1 Table — (DOCX) [file pone.0164432.s005.docx]

**Supplementary Table S1 – Transmission Electron Micrograph analysis.**

|  | WT | *kbp* | N | p-value (t-test^1^) |
| --- | --- | --- | --- | --- |
| Anterior Spinal cord | *(mean±SD)* | *(mean±SD)* |  |  |
| Mauthner area (µm^2^) | 2.399±0.49 | 2.264±0.43 | 5 each | 0.657 |
| Mauthner perimeter (µm) | 8.016±2.01 | 7.050±0.61 | 5 each | 0.333 |
| Average ventral^2, 3^ axon area (µm^2^) | 0.274±0.04 | 0.239±0.02 | 5 each | 0.124 |
| Average dorsal^3^ axon area (µm^2^) | 0.204±0.06 | 0.210±0.02 | 5 each | 0.821 |
| # Myelinated ventral axons | 7±3 | 5±2 | 5 each | 0.188 |
| # Myelinated dorsal axons | 4±2 | 3±2 | 5 each | 0.403 |
|  |  |  |  |  |
| Posterior Spinal cord | *(mean±SD)* | *(mean±SD)* |  |  |
| Average ventral axon^3^ area (µm^2^) | 0.247±0.03 | 0.214±0.06 | 5 each | 0.292 |
| Average dorsal axon^3^ area (µm^2^) | 0.338±0.11 | 0.336±0.05 | 5 each | 0.962 |

^1^ – two-tailed unpaired Student’s t-test.
^2^ – excludes Mauthner axon.
^3^ – refers to large-calibre, >0.3µm diameter axons.
