# Supplementary material for: Oligodendrocyte Development in the Absence of Their Target Axons In Vivo
Source: PLoS One. 2016 Oct 7;11(10):e0164432. doi: 10.1371/journal.pone.0164432 (PMC5055324; doi:10.1371/journal.pone.0164432)
Supplement: S2 Table — (DOCX) [file pone.0164432.s006.docx]

**Supplementary Table S2 – Time-lapse analysis – total number of events.**

|  | #mitoses | | # deaths | |
| --- | --- | --- | --- | --- |
|  | WT | *kbp* | WT | *kbp* |
| Anterior Spinal cord |  |  |  |  |
| dorsal | 23 | 7 | 0 | 0 |
| ventral | 11 | 3 | 0 | 1 |
| total | 34 | 10 | 0 | 1 |
| T (h) | 197 | 130 | 197 | 130 |
| N (larvae) | 18 | 14 | 18 | 14 |
|  |  |  |  |  |
| Posterior Spinal cord |  |  |  |  |
| dorsal | 10 | 3 | 0 | 1 |
| ventral | 9 | 5 | 0 | 12 |
| total | 19 | 8 | 0 | 13 |
| T (h) | 176 | 218 | 176 | 218 |
| N (larvae) | 18 | 21 | 18 | 21 |
